# Practice patterns and outcomes for patients with node-negative hormone receptor-positive breast cancer and intermediate 21-gene Recurrence Scores

**DOI:** 10.1186/s13058-018-0957-3

**Published:** 2018-04-16

**Authors:** Jonathan Chen, Xian Wu, Paul J. Christos, Silvia Formenti, Himanshu Nagar

**Affiliations:** 1 Department of Radiation Oncology, NewYork-Presbyterian Hospital—Weill Cornell Medicine, 525 East 68th Street, New York, NY USA; 2 Department of Healthcare Policy & Research, NewYork-Presbyterian Hospital—Weill Cornell Medicine, 525 East 68th Street, New York, NY USA

**Keywords:** Breast cancer, Chemotherapy, Intermediate risk, Recurrence Score, National Cancer Database

## Abstract

**Background:**

The recommendation for chemotherapy in early-stage breast cancer patients has been refined by the 21-gene Recurrence Score. However, uncertainty remains whether patients in the Intermediate Risk category benefit from chemotherapy.

**Methods:**

We analyzed female patients from the National Cancer Database from 2006 to 2012 who had pT1c-T2N0M0 breast cancer, were ER/PR-positive and HER2-negative, received endocrine therapy, and had a 21-gene Recurrence Score from 11 to 25. We performed univariate and multivariate logistic regression analyses to see what impacted chemotherapy receipt. We compared overall survival using Kaplan–Meier curves and the log-rank test. A multivariable Cox proportional hazards regression model was used to assess what variables impacted overall survival.

**Results:**

Of 21,991 patients who met all inclusion and exclusion criteria, 4646 (21.1%) received chemotherapy and 17,345 (78.9%) did not. Chemotherapy was more often received by patients who were younger (adjusted odds ratios (aORs) compared to age < 40 years, 0.48 for 40s, 0.34 for 50s, 0.20 for 60s, 0.10 for 70s, and 0.07 for 80+), had private insurance vs Medicare (aOR = 1.37), were from metro vs urban counties (aOR = 1.15), and were treated in community cancer centers vs academic programs (aOR = 1.26), and those with tumors of higher grade (grade 2 vs 1, aOR = 1.72; grade 3 vs 1, aOR = 3.76), higher tumor stage (pT2 vs pT1c, aOR = 1.62), or presence of lymphovascular invasion (LVI) (aOR = 1.41). At a median follow-up of 46.4 months, there was no significant difference in overall survival between patients who received chemotherapy vs those who did not (5-year estimated overall survival, 97.4% vs 97.8%, *p* = 0.89). On multivariable analysis, worse overall survival was associated with Black race, treatment at a community program, Medicaid, high-grade tumors, pT2 vs pT1c, higher Charlson–Deyo score, and no radiotherapy. Utilization trends showed that chemotherapy receipt in these patients has been decreasing from 25.8% in 2010 to 18.4% in 2013 (*p* < 0.001).

**Conclusions:**

In these patients where the benefit of chemotherapy remains uncertain, current practices see chemotherapy more likely to be used in patients with younger age, higher pathologic T stage, higher grade tumors, and LVI. No apparent difference was seen in overall survival between those who received chemotherapy and those who did not.

**Electronic supplementary material:**

The online version of this article (10.1186/s13058-018-0957-3) contains supplementary material, which is available to authorized users.

## Background

One of the pillars in cancer treatment, chemotherapy has played a significant role in improving the outcomes of breast cancer patients over the years. Traditionally, chemotherapy was offered to premenopausal women with early-stage node-negative breast cancer based primarily on tumor size (e.g., > 1 cm) and receptor status (e.g., triple-negative or HER2-positive) [1–4].

A paradigm shift in the recommendation for adjuvant chemotherapy was enabled by the advent of the Oncotype DX Recurrence Score (Genomic Health Inc., Redwood City, CA, USA), a 21-gene assay that was validated to predict the rate of distant metastases [5], the risk of breast cancer-related mortality [6], and the benefit of chemotherapy [7] in hormone receptor-positive, node-negative early-stage breast cancer patients treated with endocrine therapy. Use of this assay is now recommended by the American Society of Clinical Oncology [8] and the National Comprehensive Cancer Network guidelines [9]. The original score thresholds were chosen based on the results of the NSABP B-20 trial [5], and generated common guidelines in directing the decision to forego chemotherapy in patients with a Low Risk score (defined originally as 0–17) [10] and to recommend chemotherapy in patients with a High Risk score (originally defined as 31–100). Studies show that approximately one-third of treatment decisions were changed by the use of the Recurrence Score, and that it is a phenomenon with worldwide penetrance [11–16]. Overwhelmingly, the changes seen were in reducing the recommendation for chemotherapy in as many as half of patients [14, 17].

Subsequently published reports confirmed the safety of omitting chemotherapy in those with a Low Risk score [18]. Initial results from the low-risk arm of the Trial Assigning Individualized Options for Treatment (TAILORx trial) (21-gene Recurrence Score 0–10) recently published with a median follow-up of 69 months show excellent rates of invasive disease-free survival (93.8%) and overall survival (98.0%) [19]. However, less clarity remains for patients with an Intermediate Risk score, defined originally as a score from 18 to 30. In the analysis of the NSABP B-20 trial, the benefit of chemotherapy was clear in the High Risk group, with 10-year distant recurrence-free rates improved from 60% to 88% as well as superior overall survival; however, the addition of adjuvant chemotherapy failed to result in significantly better distant recurrence-free rates (91% vs 89%) or overall survival in the Intermediate Risk group [7].

The currently ongoing TAILORx trial is a prospective randomized trial studying whether patients with Oncotype DX Intermediate Risk scores benefit from chemotherapy [10, 20]. Of note, the investigators shifted the thresholds defining the risk groups to be more conservative, with Intermediate Risk defined as a score of 11–25, to minimize the risk of undertreatment. In the NSABP B-20 trial, a Recurrence Score of 11 was associated with a distant recurrence risk of 10%, a commonly used threshold for recommending adjuvant chemotherapy [10].

Until the results of the TAILORx trial are reported, we rely on retrospective data to guide adjuvant chemotherapy recommendations in women with Oncotype DX scores of 11–25. A recent retrospective review from MD Anderson Cancer Center at a median follow-up of 58 months reported no additional benefit from adjuvant chemotherapy (invasive DFS, RFS, DRFS, and OS) among 894 patients with hormone receptor-positive, HER2-negative, lymph node-negative, early-stage breast cancer with an Oncotype DX Recurrence Score of 11–25 treated at their institution [21]. Another retrospective analysis of a prospectively designed registry showed excellent outcomes for this same population without chemotherapy [22].

We queried the National Cancer Database (NCDB) for practice patterns and survival outcomes in node-negative T1c-T2 female breast cancer patients with hormone receptor-positive/HER2-negative tumors and Oncotype DX Recurrence Score of 11–25, with a focus on the use of adjuvant chemotherapy.

## Methods

### Data source

The NCDB is a joint project of the American College of Surgeons and the American Cancer Society that draws data from more than 1500 accredited cancer programs accounting for 70% of all newly diagnosed cancer cases in the USA (National Cancer Institute, Bethesda, MD, USA).

### Patient selection

**Fig. 1:**
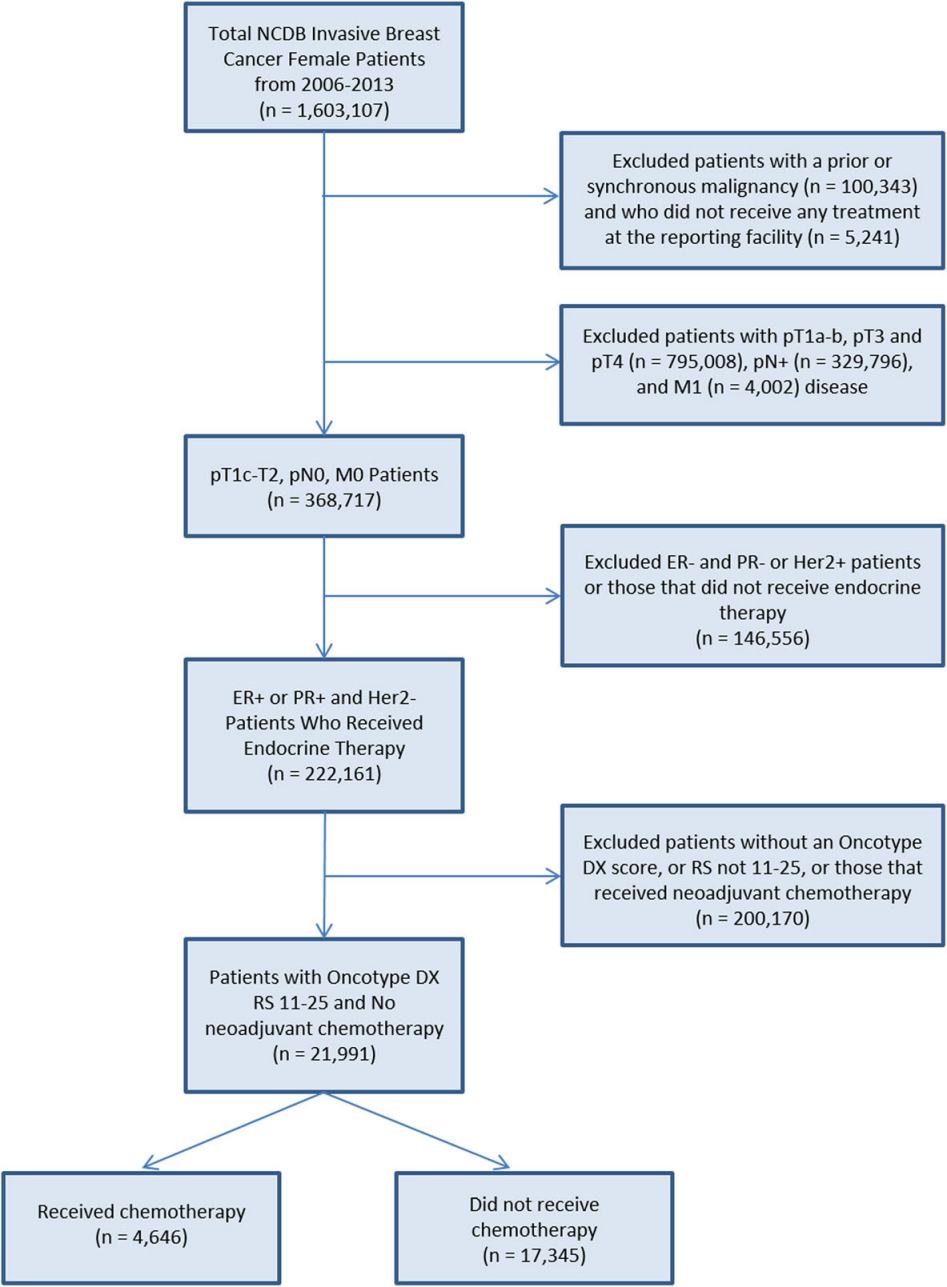
Consolidated Standards of Reporting Trials (CONSORT) diagram showing inclusion and exclusion criteria for this study. ER estrogen receptor, Her2 human epidermal growth factor receptor 2, NCDB National Cancer Database, PR progesterone receptor, RS Recurrence Score

Patients were selected to mimic the criteria of the TAILORx trial. Women included in our analysis had pathologic stage T1c-T2N0M0 (AJCC 6th edition, 2004+) breast cancer, ER-positive or PR-positive and HER2-negative receptor status, and an Oncotype DX score from 11 to 25, diagnosed between 2006 and 2012. Only women who received endocrine therapy were included. Exclusion criteria included patients who received neoadjuvant chemotherapy. In total, 21,991 cases were included in the analysis. These criteria are displayed in a CONSORT diagram in Fig. 1.

### Definition of variables

Patients and treatment characteristics included facility type, age, ethnicity, insurance type, median income, education**,** geographic location, Charlson–Deyo comorbidity score, year of diagnosis, breast cancer laterality, grade, clinical and pathologic tumor size, clinical and pathologic stage, presence of lymphovascular invasion (LVI), type of surgery, surgical margin status, receipt of adjuvant radiotherapy and/or chemotherapy, and nodal irradiation.

### Statistical analysis

Descriptive statistics (including mean, standard deviation, median, range, frequency, and percentage) were calculated to characterize the study cohort. Demographic, prognostic, and facility characteristics (all categorical variables) were compared between patients who received chemotherapy vs those who did not receive chemotherapy by the chi-square test. Univariate and multivariable logistic regression analyses were employed to determine factors independently associated with receipt of chemotherapy. Factors of interest included facility type, age, race/ethnicity, primary payer, urban/rural location, Charlson–Deyo comorbidity score, grade, AJCC pathologic T stage, LVI, type of surgery, surgical margin status, and radiotherapy status. To evaluate the impact of chemotherapy on all-cause mortality, we first plotted Kaplan–Meier curves and evaluated differences in overall survival between categories of interest with the log-rank test. We then constructed a multivariable Cox proportional hazards regression model for all-cause mortality, adjusting for the same demographic, prognostic, and facility characteristics already listed as these variables were all of interest a priori. Hazard ratios (HRs) and 95% confidence intervals (CIs) were reported. The proportional hazards assumption was examined using Schoenfeld residuals and it was not violated. We also separately analyzed those patients who were diagnosed between 2006 and 2010 to examine a subset with more meaningful length of follow-up. All statistical tests were two-sided, with statistical significance evaluated at the 0.05 alpha level. All analyses were conducted in SAS version 9.4 (SAS Institute, Inc., Cary, NC, USA).

## Results

There were 21,991 patients from the NCDB who met our inclusion and exclusion criteria. From this group, 4646 (21.1%) received chemotherapy and 17,345 (78.9%) did not. Table 1 presents all variables analyzed and compares them based on chemotherapy use via univariate analysis.

**Table 1:** Patient demographics and characteristics

	All patients^a^ (*N* = 21,991)		No chemotherapy^b^ (*N* = 17,345)		chemotherapy^b^ (*N* = 4646)		
Characteristic	*n*	%	*n*	%	*n*	%	*p* value^c^
Facility type							< 0.001
Community cancer program	1914	8.7	1504	8.7	410	8.8	
Comprehensive community cancer program	10,435	47.5	8379	48.3	2056	44.3	
Academic/research program	7172	32.6	5666	32.7	1506	32.4	
Other	2470	11.2	1796	10.4	674	14.5	
Age category (years)							< 0.001
< 40	747	3.4	390	2.3	357	7.7	
40–49	4735	21.5	3287	19.0	1448	31.2	
50–59	7014	31.9	5380	31.0	1634	35.2	
60–69	6823	31.0	5810	33.5	1013	21.8	
70–79	2469	11.2	2288	13.2	181	3.9	
80+	203	0.9	190	1.1	13	0.3	
Race/ethnicity							0.004
Non-Hispanic White	17,628	80.2	13,982	80.6	3646	78.5	
Non-Hispanic Black	1535	7.0	1189	6.9	346	7.5	
Hispanic	898	4.1	685	4.0	213	4.6	
Non-Hispanic Asian/Pacific	761	3.5	569	3.3	192	4.1	
Other	1169	5.3	920	5.3	249	5.4	
Primary payer							< 0.001
Not insured	341	1.6	248	1.4	93	2.0	
Private insurance	14,567	66.2	10,994	63.4	3573	76.9	
Medicaid	1172	5.3	881	5.1	291	6.3	
Medicare	5479	24.9	4880	28.1	599	12.9	
Other	432	2.0	342	2.0	90	1.9	
Urban/rural							0.62
Metro counties	18,447	83.9	14,531	83.8	3916	84.3	
Urban counties	2657	12.1	2120	12.2	537	11.6	
Rural counties	354	1.6	275	1.6	79	1.7	
Unknown	533	2.4	419	2.4	114	2.5	
Charlson–Deyo score							< 0.001
0	19,070	86.7	14,980	86.4	4090	88.0	
1	2540	11.6	2035	11.7	505	10.9	
≥ 2	381	1.7	330	1.9	51	1.1	
Grade							< 0.001
Well differentiated, differentiated, NOS	5723	26.0	4971	28.7	752	16.2	
Moderately differentiated, moderately well differentiated, intermediate differentiation	12,305	56.0	9709	56.0	2596	55.9	
Poorly differentiated	2780	12.6	1751	10.1	1029	22.2	
Undifferentiated, anaplastic	13	0.1	8	0.1	5	0.1	
Cell type not determined, not stated or not applicable, unknown primaries, high-grade dysplasia	1170	5.3	906	5.2	264	5.7	
AJCC pathologic stage							< 0.001
T1C	15,766	71.7	12,827	74.0	2939	63.3	
T2	6225	28.3	4518	26.1	1707	36.7	
Lymphovascular invasion							< 0.001
Not present	17,155	78.0	13,795	79.5	3360	72.3	
Present	2199	10.0	1507	8.7	692	14.9	
Not applicable	14	0.1	10	0.1	4	0.1	
Unknown	2623	11.9	2033	11.7	590	12.7	
Type of surgery							< 0.001
Breast conservation	15,468	70.3	12,426	71.6	3042	65.5	
Mastectomy	6521	29.7	4919	28.4	1602	34.5	
Unknown	2	0.01	0	0.0	2	0.04	
Margin status							0.01
Negative	21,189	96.4	16,745	96.5	4444	95.7	
Positive	725	3.3	544	3.1	181	3.9	
Unknown	77	0.4	56	0.3	21	0.5	
Radiation therapy							< 0.001
None	6557	29.8	5032	29.0	1525	32.8	
Beam radiation	14,116	64.2	11,183	64.5	2933	63.1	
Other/unknown	1318	6.0	1130	6.5	188	4.1	
Radiation treatment volume							< 0.001
No radiation treatment	6556	29.8	5031	29.0	1525	32.8	
Breast or chest wall	14,829	67.4	11,875	68.5	2954	63.6	
Breast/lymph nodes or chest wall/lymph nodes	470	2.1	350	2.0	120	2.6	
Other	53	0.2	36	0.2	17	0.4	
Unknown	83	0.4	53	0.3	30	0.7	

*AJCC* American Joint Committee on Cancer, *NOS* not otherwise specified

^a^Characteristics for all patients who met inclusion criteria for this study

^b^Comparison of characteristics for the patients who did not receive chemotherapy to the patients who did receive chemotherapy

^c^Differences between these two populations on univariate analysis

With regards to practice patterns in the USA, chemotherapy was more often received by patients with the following characteristics on multivariable analysis (Table 2): younger age (all adjusted odds ratios (aORs) for age categories are compared to age < 40 years, 0.48 for 40s, 0.34 for 50s, 0.20 for 60s, 0.10 for 70s, and 0.07 for 80+; all *p* < 0.001), those treated in community cancer centers (aOR = 1.26, *p* = 0.01) or comprehensive community centers (aOR = 1.20, *p* < 0.001) vs academic programs, those in metro counties vs urban counties (aOR = 1.15, *p* = 0.05), and those privately insured vs insured by Medicare (aOR = 1.37, *p* < 0.001). Tumor characteristics that were associated with greater chemotherapy use were pT2 vs pT1c (aOR = 1.62, *p* < 0.001), higher pathological grade (grade 2 vs 1, aOR = 1.72, *p* < 0.001; grade 3 vs 1, aOR = 3.76, *p* < 0.001), and presence of LVI (aOR = 1.41, *p* < 0.001). The type of surgery was also associated with chemotherapy use, with more frequent use among patients who underwent mastectomy vs breast conservation surgery (aOR = 1.35, *p* = 0.02).

**Table 2:** Multivariable analysis of patient demographics and characteristics comparing patients who did not receive chemotherapy to patients who did receive chemotherapy

	Adjusted odds ratio	95% confidence interval	*p* value
Facility type			
Community cancer program	1.26	1.07–1.49	0.01
Comprehensive community cancer program	1.20	1.09–1.32	< 0.001
Academic/research program	REF		
Other	1.13	0.95–1.33	0.16
Age category (years)			
< 40	REF		
40–49	0.48	0.38–0.61	< 0.001
50–59	0.34	0.27–0.43	< 0.001
60–69	0.20	0.16–0.26	< 0.001
70–79	0.10	0.07–0.14	< 0.001
80+	0.07	0.03–0.16	< 0.001
Race/ethnicity			
Non-Hispanic White	REF		
Non-Hispanic Black	1.10	0.94–1.40	0.26
Hispanic	1.04	0.85–1.27	0.73
Non-Hispanic Asian/Pacific	0.94	0.76–1.17	0.60
Other	1.15	0.94–1.40	0.19
Primary payer			
Not insured	1.09	0.80–1.48	0.59
Private insurance	REF		
Medicaid	0.91	0.76–1.09	0.30
Medicare	0.73	0.63–0.85	< 0.001
Other	0.79	0.58–1.08	0.14
Urban/rural			
Metro counties	REF		
Urban counties	0.87	0.76–1.00	0.05
Rural counties	1.30	0.95–1.79	0.10
Unknown	0.84	0.63–1.12	0.22
Charlson–Deyo score			
0	REF		
1	1.09	0.95–1.24	0.22
2	0.73	0.49–1.08	0.11
Grade			
Well differentiated, differentiated, NOS	REF		
Moderately differentiated, moderately well differentiated, intermediate differentiation	1.72	1.54–1.91	< 0.001
Poorly differentiated	3.76	3.28–4.31	< 0.001
AJCC pathologic stage			
T1C	REF		
T2	1.62	1.48–1.77	< 0.001
Lymphovascular invasion			
Not present	REF		
Present	1.41	1.25–1.59	< 0.001
Type of surgery			
Breast conservation	REF		
Mastectomy	1.35	1.06–1.72	0.02
Margin status			
Negative	REF		
Positive	1.15	0.92–1.44	0.23
Unknown	1.05	0.45–2.46	0.90
Radiation therapy			
None	REF		
Beam radiation	0.35	0.01–8.40	0.51
Radiation treatment volume			
No radiation treatment	0.25	0.01–6.20	0.40
Breast or chest wall	REF		
Breast/lymph nodes or chest wall/lymph nodes	1.24	0.96–1.61	0.10
Other	2.36	1.05–5.30	0.04
Unknown	1.30	0.56–3.04	0.54

*AJCC* American Joint Committee on Cancer, *NOS* not otherwise specified, *REF* reference

As stated earlier, younger women received chemotherapy more often in this study when age groups were divided into decades. We also performed univariate analysis on chemotherapy use among women aged younger than 35, 35–50, and > 50 years. In these age groups, 53.2%, 31.3%, and 16.8% received chemotherapy, respectively (*p* < 0.001). These data are presented in Additional file 1: Table S1. Age thresholds of 35 and 50 years were selected to correspond to clinically relevant ages of young breast cancer patients and postmenopausal patients. Factors that did not significantly correlate with the receipt of chemotherapy on multivariable analysis included the Charlson–Deyo comorbidity score and margin status.

**Fig. 2:**
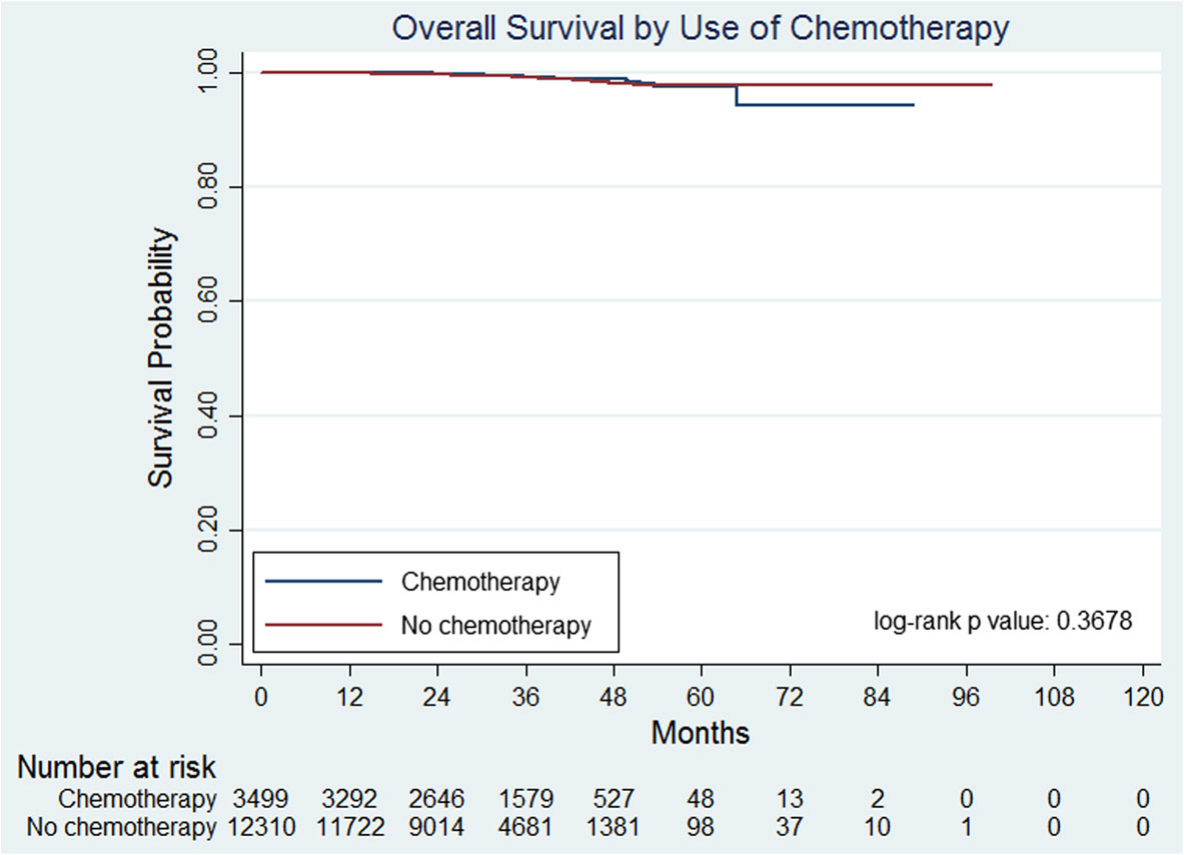
Overall survival of patients (2006–2010) who received chemotherapy did not differ significantly from patients who did not receive chemotherapy in this study, with estimated 5-year overall survival of 97.4% vs 97.8% at a median follow-up of 46.4 months (*p* = 0.89)

Kaplan–Meier overall survival curves were generated. At a median follow-up of 32.1 months, there was no significant difference in overall survival between patients who received chemotherapy vs those who did not (*p* = 0.37, Fig. 2). At 5 years, the estimated overall survival was 97.4% (95% CI 95.3–98.5%) in the group that received chemotherapy vs 97.6% (95% CI 96.9–98.2%) in the group that did not receive chemotherapy (adjusted HR = 0.83, 95% CI 0.55–1.25). Of the patients included in this study, 4753 were diagnosed between 2006 and 2010. Kaplan–Meier overall survival curves were also generated for this subgroup (Additional file 2: Figure S1). With a longer median follow-up of 46.4 months, there was also no significance difference in overall survival in this subset between patients who received chemotherapy vs those who did not (*p* = 0.89).

On multivariable analysis, the following variables were associated with worse overall survival (Table 3): treatment at a community program vs an academic program (adjusted HR = 2.44, *p* = 0.01), Black vs White race (adjusted HR = 1.99, *p* = 0.02), Medicaid vs private insurance (adjusted HR = 2.21, *p* = 0.05), poorly differentiated vs well differentiated tumor (adjusted HR = 2.00, p = 0.02), pathologic T2 vs T1c (adjusted HR = 2.17, *p* < 0.001), Charlson–Deyo score of 1 (adjusted HR = 3.00, p < 0.001) and score of 2 (adjusted HR = 6.74, p < 0.001) vs score of 0, and no radiotherapy vs radiotherapy (adjusted HR = 2.67, *p* = 0.02). Conversely, the margin status, LVI, and type of surgery had no significant associations with survival outcome.

**Table 3:** Multivariable analysis of patient demographics and characteristics comparing overall survival

	Adjusted hazard ratio	95% confidence interval	*p* value
Facility type			
Community cancer program	2.44	1.26–4.75	0.01
Comprehensive community cancer program	1.54	0.92–2.58	0.10
Academic/research program	REF		
Other	1.45	0.66–3.17	0.36
Age category (years)			
< 40	REF		
40–49	1.41	0.16–12.31	0.76
50–59	2.36	0.29–19.42	0.43
60–69	2.56	0.31–21.28	0.38
70–79	5.02	0.58–43.63	0.14
80+	8.40	0.89–89.81	0.08
Race/ethnicity			
Non-Hispanic White	REF		
Non-Hispanic Black	1.99	1.12–3.52	0.02
Hispanic	0.51	0.12–2.11	0.36
Non-Hispanic Asian/Pacific	0.86	0.21–3.56	0.84
Other	0.46	0.14–1.44	0.18
Primary payer			
Not insured	1.78	0.43–7.41	0.43
Private insurance	REF		
Medicaid	2.21	1.01–4.80	0.05
Medicare	1.38	0.77–2.48	0.28
Other	2.76	0.97–7.87	0.06
Urban/rural			
Metro counties	REF		
Urban counties	1.04	0.60–1.82	0.89
Rural counties	1.78	0.55–5.76	0.33
Unknown	0.76	0.18–3.14	0.70
Charlson–Deyo score			
0	REF		
1	3.00	1.92–4.67	< 0.001
2	6.74	3.40–13.34	< 0.001
Grade			
Well differentiated, differentiated, NOS	REF		
Moderately differentiated, moderately well differentiated, intermediate differentiation	1.13	0.69–1.87	0.63
Poorly differentiated	2.00	1.10–3.63	0.02
AJCC pathologic stage			
T1C	REF		
T2	2.17	1.45–3.25	< 0.001
Lymphovascular invasion			
Not present	REF		
Present	1.23	0.70–2.16	0.48
Type of surgery			
Breast conservation	REF		
Mastectomy	0.45	0.20–1.02	0.06
Radiation therapy			
None	REF		
Beam radiation	0.38	0.17–0.83	0.01

*AJCC* American Joint Committee on Cancer, *NOS* not otherwise specified, *REF* reference

**Fig. 3:**
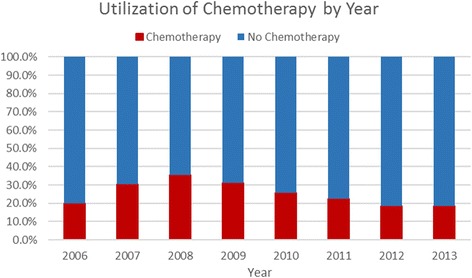
Utilization of chemotherapy by year. Data from NCDB show that chemotherapy use in the patients in this study has decreased every year from 2008 to 2013, although sample size was limited until 2010 (*n* = 62 in 2008, *n* = 290 in 2009, and *n* = 4377 in 2010). This decrease was statistically significant (for trend from 2010 to 2013, *p* < 0.001)

Figure 3 and Additional file 3: Table S2 show chemotherapy utilization trends stratified by year. The absolute number of patients available from 2006 and 2007 is small (*n* = 5 and *n* = 23, respectively), and only starting in 2010 were a significant number of cases captured by the database. A decrease in chemotherapy utilization in this patient population was reported from 25.8% in 2010 to 18.4% in 2013 (*p* for trend < 0.001).

**Fig. 4:**
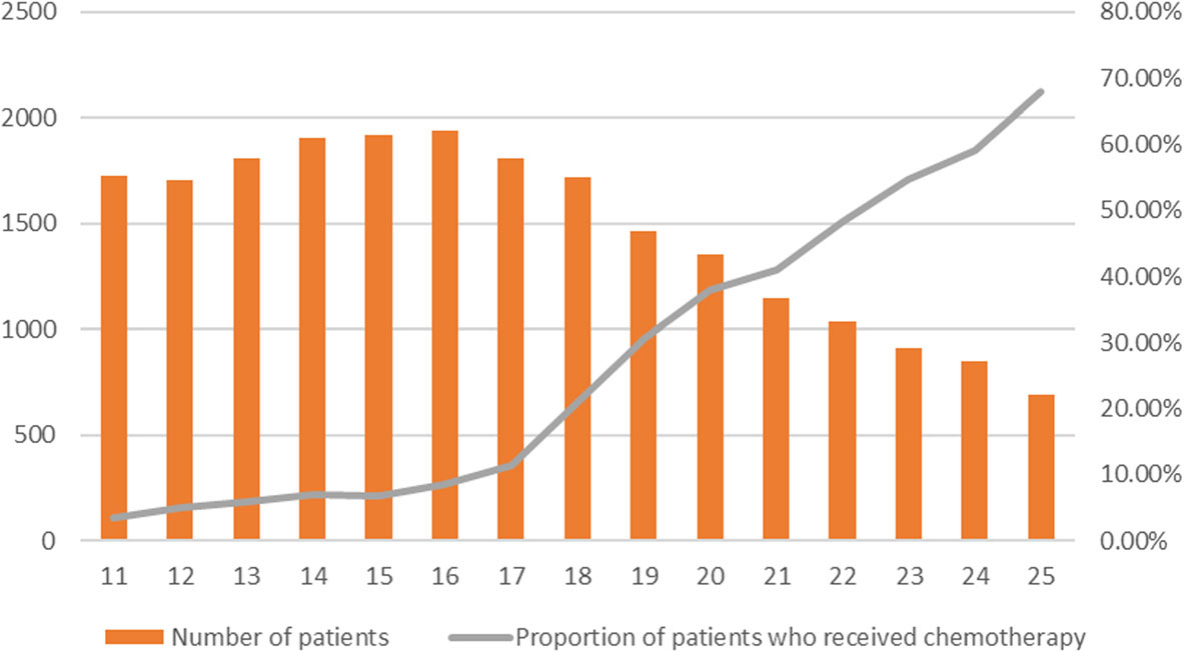
Incidence of individual 21-gene Recurrence Scores from 11 to 25, and comparative utilization of chemotherapy based on the score. Most patients in this range are in the lower half (score 11–18). Chemotherapy use in patients sees a dramatic increase starting at a score of 18, and rises steadily for each point increase

Figure 4 and Additional file 4: Table S3 show chemotherapy utilization trends stratified by Recurrence Score, as well as the absolute incidence of patients with each score. In the specific population of breast cancer patients in this study, there appears to be a higher incidence of Recurrence Scores in the lower half of the range than the upper half. The rate of chemotherapy utilization increased steadily with increasing Recurrence Score, after a sharp increase in rate seen at a score above 17.

## Discussion

Our findings indicate that among 21,991 hormone receptor-positive, HER2-negative, early-stage node-negative breast cancer patients who received endocrine therapy and had a 21-gene Recurrence Score of 11–25, chemotherapy was more often given to patients (on multivariate analysis) of younger age, with private insurance, not treated at academic programs, from metro counties, and whose tumors had higher pathologic T stage, higher grade, or LVI.

It is not surprising that younger age, pathologic T stage, LVI, and higher grade correlated with increased chemotherapy use as all are recognized as signs of more aggressive cancers that confer a higher risk of recurrence. Therefore, these risk factors could have justified the choice for chemotherapy regardless of the Oncotype DX Recurrence Score.

Margin status was not correlated with chemotherapy use; this factor is generally considered to be more indicative of risk for local recurrence. Accordingly, in our patient population, positive margins were significantly correlated with adjuvant RT (79.8% receiving RT vs 69.9%).

Two socioeconomic factors associated with chemotherapy use were type of insurance and type of facility. Patients with private insurance were more likely to receive chemotherapy than those with Medicare, but there was no difference compared to patients with Medicaid. Since we have evidence that age was significantly correlated with chemotherapy use, the most obvious explanation was the age distribution of patients with private insurance vs Medicare. Indeed, when we repeated multivariate analysis only with patients over the age of 65 years, there was no longer any statistical difference in chemotherapy use between any insurance groups.

Chemotherapy was given less often at academic programs compared to nonacademic programs. It is possible that doctors in the community may be more conservative, opting for more aggressive treatment. Alternatively, their patients may have presented with more advanced disease warranting a greater use of chemotherapy. In our study population, there was no difference in pathologic T stage between academic and nonacademic programs, but there may be a difference in other risk factors not captured in the database like multicentric disease. Furthermore, if nonacademic programs saw patients with worse disease, one might expect a greater rate of mastectomies, but this was not the case (data not shown).

In the multivariable analysis of overall survival in these patients, it was alarming to see significantly worse survival in patients treated in the community, those with Medicaid, and those of Black race. These are potentially indicative of imbalances in access to care in our healthcare system.

Our findings also indicate that chemotherapy use has been steadily decreasing in this patient population from 2010 to 2013, perhaps signifying a shift in thinking concerning the need for chemotherapy in this Intermediate Risk group. Additionally, our findings show a clear relationship between Oncotype DX Recurrence Score and chemotherapy use, with a sharp rise in chemotherapy use starting at a score of 18, the original threshold used to distinguish low-risk and intermediate-risk patients, and a linear increase in chemotherapy use from 18 to 25.

Our analysis demonstrates comparable outcomes in patients who received chemotherapy vs those who did not, at a median follow-up of 32 months. This lack of difference persisted when we only analyzed patients from 2006 to 2010, for a longer median follow-up of 46 months. These findings are consistent with the results seen in the initial analysis of the NSABP B-20 trial [7]. However, survival outcomes from retrospective analyses of large databases like the NCDB require cautious interpretation. First, survival outcomes in early-stage breast cancer require 10 years or more of follow-up for informative results. The NCDB started accumulating data in 1989, but Oncotype DX data were not adequately collected until 2006; more time is required for these data to mature.

Most importantly, well-known limitations pertain to all retrospective database studies. These types of studies are intrinsically limited in their ability to reveal causal relationships, especially regarding survival endpoints. Selection biases assigning patients to specific treatments are often impossible to identify, and these biases generate confounding variables when defining the causal effect of an intervention. Despite our efforts to take most variables into consideration, other doctors’ or patients’ related preferences may have exerted an effect not captured in the database. For example, physicians might have used their clinical judgment to preferentially give chemotherapy to patients at higher risk for recurrence, distant metastasis, and/or breast cancer-related mortality using risk factors not recorded in the NCDB. Consistent with this hypothesis, in this study patients who underwent mastectomy were more likely to also receive chemotherapy. It is possible that the treating physicians perceived these patients as higher risk, and consequently recommended more aggressive therapies that included mastectomy, nodal RT, and chemotherapy. One cannot exclude that the addition of chemotherapy in these cases may have mitigated the negative prognostic impact of these risks and in fact reduced the likelihood of recurrence among these recipients, resulting in comparable outcomes.

Only prospective studies randomly assigned to chemotherapy vs no chemotherapy can generate evidence that avoids these biases. Comparisons of results from prospective randomized trials with those from retrospective analysis asking the same question can result in widely dissimilar findings. For example, the effect of radiation therapy on the outcome of breast cancer patients from the Early Breast Cancer Trialists’ Collaborative Group Meta-Analyses drastically differed from a report based on the SEER registries [23].

Two other limitations of our study include excluding patients who did not receive endocrine therapy, and dissimilarities between our patient population and those being studied in the TAILORx trial. By removing patients who did not receive endocrine therapy, we may have skewed our results by excluding women with more comorbidities, poorer compliance, and so forth. While our study was designed to predominantly match the criteria of the TAILORx trial, deviations include no exclusion of patients with COPD, chronic liver disease, CVA, CHF or other heart disease, and chronic psychiatric conditions (although we attempted to partly compensate by including the Charlson–Deyo comorbidity score in our analysis); inability to assess life expectancy and therefore not excluding patients with < 10-year life expectancy; and not including high-risk pT1b or pT3 patients. Querying the NCDB for pT3N0M0 patients who also met all other inclusion/exclusion criteria used in this study yielded only 281 patients. It is unlikely that the inclusion or exclusion of this small subset of patients would have had an impact on the findings of this study. Another limitation is the lack of data in the NCDB regarding recurrence, pattern of recurrence, and cause of death. This limits the outcomes we are able to evaluate.

Lastly, an important caveat is that the patients in this study may not be fully representative of patients in practice. In the NCDB from 2006 to 2013, 23.2% of hormone receptor-positive, HER2-negative, node-negative breast cancer patients had a documented Oncotype DX score, while 51.9% were listed as “Not Applicable: Information not collected for this case” and 8.1% were “Unknown” (data not shown). Those who were selected to undergo the test may have had higher risk disease. Furthermore, insurance may preferentially cover Oncotype DX testing for ER-positive tumors, while we included ER-negative, PR-positive tumors as well.

Our study documents the practice patterns of chemotherapy use in this select population of breast cancer patients, and highlights the importance of the Oncotype DX Recurrence Score in influencing treatment recommendations. Individualized medicine involves separating patients into specific subsets using scientific knowledge to identify meaningful parameters. The establishment of the Low, Intermediate, and High Risk groups by the Oncotype DX score is an important step in this direction. These types of tests provide more clarity for patients and healthcare providers, reduce overtreatment, and potentially reduce healthcare costs. The application of the Recurrence Score has been modeled to be an economically advantageous clinical tool, possibly saving over $1000 per patient [11, 24]. Further partitioning of Low, Intermediate, and High Risk groups of patients will optimize therapy for each individual.

To that end, other genetic panels are also being used including the Mammaprint 70-gene recurrence assay (Agendia, Irvine, CA, USA) and the PAM-50 (also known as Prosigna Breast Cancer Prognostic Gene Signature Assay; NanoString Technologies, Seattle, WA, USA). The Mammaprint assay has shown promise in identifying early-stage breast cancer patients with zero to three positive lymph nodes who may not need chemotherapy [25]. Interestingly, one of the selling points of the Mammaprint assay is that it is binary in classifying patients as Low Risk or High Risk, eliminating the ambiguity of an Intermediate Risk result [26]. Preliminary results also show potential for Mammaprint results to substratify Oncotype DX Intermediate Risk patients [27]. The growing importance of these genetic panels is highlighted by their inclusion in the eighth edition of the AJCC staging guidelines [28].

## Conclusions

The additional benefit of adding chemotherapy in early-stage hormone receptor-positive breast cancer patients remains to be fully defined. While doctors’ and patients’ choice for additional chemotherapy may have mitigated risks associated with some higher-risk patients, this early report from the NCDB suggests that patients with an Oncotype DX score of 11–25 who received chemotherapy had a comparable survival to those who did not. The results of the prospective randomized TAILORx trial will provide evidence concerning the role of adjuvant chemotherapy in intermediate-risk early breast cancer and help reduce overtreatment.

## Additional files

Additional file 1: **Table S1.** Chemotherapy receipt by age group, with age separated into three tiers using 35 and 50 years as clinically relevant thresholds. Chemotherapy receipt was significantly dependent on age group in this study (chi-square test, *p* < 0.001). (DOCX 15 kb)

Additional file 2: **Figure S1.** No difference in  overall survival by use of chemotherapy for patients diagnosed between 2006 and 2010 with median follow-up of 46.4 months. (TIFF 150 kb)

Additional file 3: **Table S2.** Utilization of chemotherapy by year. Chemotherapy use has been decreasing steadily over the years (trend from 2010 to 2013, *p* < 0.001). (DOCX 14 kb)

Additional file 4: **Table S3.** Incidence of individual 21-gene Recurrence Scores from 11 to 25, and the comparative utilization of chemotherapy based on the score. (DOCX 14 kb)

## References

[1] Chen C, Dhanda R, Tseng WY (2013). Evaluating use characteristics for the Oncotype DX 21-gene recurrence score and concordance with chemotherapy use in early-stage breast cancer. J Oncol Pract.

[2] Eifel P, Axelson JA, Costa J (2001). National Institutes of Health Consensus Development Conference Statement: adjuvant therapy for breast cancer, November 1–3, 2000. J Natl Cancer Inst.

[3] Goldhirsch A, Wood WC, Gelber RD (2003). Meeting highlights: updated international expert consensus on the primary therapy of early breast cancer. J Clin Oncol.

[4] Clarke M (2006). Meta-analyses of adjuvant therapies for women with early breast cancer: the Early Breast Cancer Trialists’ Collaborative Group overview. Ann Oncol.

[5] Paik S, Shak S, Tang G (2004). A multigene assay to predict recurrence of tamoxifen-treated, node-negative breast cancer. N Engl J Med.

[6] Habel LA, Shak S, Jacobs MK (2006). A population-based study of tumor gene expression and risk of breast cancer death among lymph node-negative patients. Breast Cancer Res.

[7] Paik S, Tang G, Shak S (2006). Gene expression and benefit of chemotherapy in women with node-negative, estrogen receptor-positive breast cancer. J Clin Oncol.

[8] Harris LN, Ismaila N, McShane LM (2016). American Society of Clinical Oncology. Use of biomarkers to guide decisions on adjuvant systemic therapy for women with early-stage invasive breast cancer: American Society of Clinical Oncology Clinical Practice Guideline. J Clin Oncol.

[9] Gradishar WJ, Anderson BO, Balassanian R (2016). Invasive Breast Cancer Version 1.2016, NCCN Clinical Practice Guidelines in Oncology. J Natl Compr Cancer Netw.

[10] Sparano J, Paik S (2008). Development of the 21-gene assay and its application in clinical practice and clinical trials. J Clin Oncol.

[11] Hornberger J, Chien R, Krebs K, Hochheiser L (2011). US Insurance Program's experience with a multigene assay for early-stage breast cancer. J Oncol Pract.

[12] Jaafar H, Bashir MA, Taher A, Qawasmeh K, Jaloudi M (2014). Impact of Oncotype DX testing on adjuvant treatment decisions in patients with early breast cancer: a single-center study in the United Arab Emirates. Asia Pac J Clin Oncol.

[13] Bargallo JE, Lara F, Shaw-Dulin R (2015). A study of the impact of the 21-gene breast cancer assay on the use of adjuvant chemotherapy in women with breast cancer in a Mexican public hospital. J Surg Oncol.

[14] Lee MH, Han W, Lee JE (2015). The clinical impact of 21-gene recurrence score on treatment decisions for patients with hormone receptor-positive early breast cancer in Korea. Cancer Res Treat.

[15] Albanell J, Svedman C, Gligorov J (2016). Pooled analysis of prospective European studies assessing the impact of using the 21-gene Recurrence Score assay on clinical decision making in women with oestrogen receptor-positive, human epidermal growth factor receptor 2-negative early-stage breast cancer. Eur J Cancer.

[16] Leung RC, Yau TC, Chan MC (2016). The impact of the Oncotype DX breast cancer assay on treatment decisions for women with estrogen receptor-positive, node-negative breast carcinoma in Hong Kong. Clin Breast Cancer.

[17] Lo SS, Mumby PB, Norton J (2010). Prospective multicenter study of the impact of the 21-gene recurrence score assay on medical oncologist and patient adjuvant breast cancer treatment selection. J Clin Oncol.

[18] Gluz O, Nitz UA, Christgen M (2016). West German Study Group Phase III PlanB Trial: first prospective outcome data for the 21-gene recurrence score assay and concordance of prognostic markers by central and local pathology assessment. J Clin Oncol.

[19] Sparano JA, Gray RJ, Makower DF (2015). Prospective validation of a 21-gene expression assay in breast cancer. N Engl J Med.

[20] Sparano JA (2006). TAILORx: trial assigning individualized options for treatment (Rx). Clin Breast Cancer.

[21] Barcenas CH, Raghavendra A, Sinha AK (2017). Outcomes in patients with early-stage breast cancer who underwent a 21-gene expression assay. Cancer.

[22] Stemmer SM, Steiner M, Rizel S (2017). Clinical outcomes in patients with node-negative breast cancer treated based on the recurrence score results: evidence from a large prospectively designed registry. NPJ Breast Cancer.

[23] Henson KE, Jagsi R, Cutter D, McGale P, Taylor C, Darby SC (2016). Inferring the effects of cancer treatment: divergent results from Early Breast Cancer Trialists’ Collaborative Group meta-analyses of randomized trials and observational data from SEER registries. J Clin Oncol.

[24] Lyman GH, Cosler LE, Kuderer NM, Hornberger J (2007). Impact of a 21-gene RT-PCR assay on treatment decisions in early-stage breast cancer: an economic analysis based on prognostic and predictive validation studies. Cancer.

[25] Cardoso F, van't Veer LJ, Bogaerts J (2016). MINDACT Investigators. 70-Gene signature as an aid to treatment decisions in early-stage breast cancer. N Engl J Med.

[26] Agendia. http://www.agendia.com/healthcare-professionals/breast-cancer/mammaprint/. Accessed 26 Jun 2017.

[27] Tsai M, Untch S, Blumencranz L, Treece T, Lo S, Soliman H (2016). The 70-gene signature to provide risk stratification and treatment guidance for patients classified as intermediate by the 21-gene assay. J Clin Oncol.

[28] Amin MB, Edge SB, Greene FL (2017). AJCC Cancer Staging Manual.

