# Supplementary material for: Practice patterns and outcomes for patients with node-negative hormone receptor-positive breast cancer and intermediate 21-gene Recurrence Scores
Source: Breast Cancer Res. 2018 Apr 16;20:26. doi: 10.1186/s13058-018-0957-3 (PMC5903005; doi:10.1186/s13058-018-0957-3)
Supplement: Supplementary file 1 — Table S1. Chemotherapy receipt by age group, with age separated into three tiers using 35 and 50 years as clinically relevant thresholds. Chemotherapy receipt was significantly dependent on age group in this study (chi-square test, p < 0.001). (DOCX 15 kb) [file 13058_2018_957_MOESM1_ESM.docx]

Additional file 1: Table S1

Table of chemotherapy receipt by age group, with age separated into three tiers using 35 and 50 as clinically relevant thresholds. Chemotherapy receipt was significantly dependent on age group in this study (Chi-square test p-value <0.001).

| **Table of Chemotherapy Receipt by Age Group** | | | |
| --- | --- | --- | --- |
| **Age** | **Received Chemotherapy** | | |
|  | **No** | **Yes** | **Total** |
| **<35** | \| 111 \| \| --- \| \| 46.8% \| | \| 126 \| \| --- \| \| 53.2% \| | \| 237 \| \| --- \| \|  \| |
| **35-50** | \| 4113 \| \| --- \| \| 68.7% \| | \| 1874 \| \| --- \| \| 31.3% \| | \| 5987 \| \| --- \| \|  \| |
| **>50** | \| 13121 \| \| --- \| \| 83.2% \| | \| 2646 \| \| --- \| \| 16.8% \| | \| 15767 \| \| --- \| \|  \| |
| **Total** | \| 17345 \| \| --- \| | \| 4646 \| \| --- \| | \| 21991 \| \| --- \| |
