# Supplementary figures and images for: Practice patterns and outcomes for patients with node-negative hormone receptor-positive breast cancer and intermediate 21-gene Recurrence Scores
Source: Breast Cancer Res. 2018 Apr 16;20:26. doi: 10.1186/s13058-018-0957-3 (PMC5903005; doi:10.1186/s13058-018-0957-3)

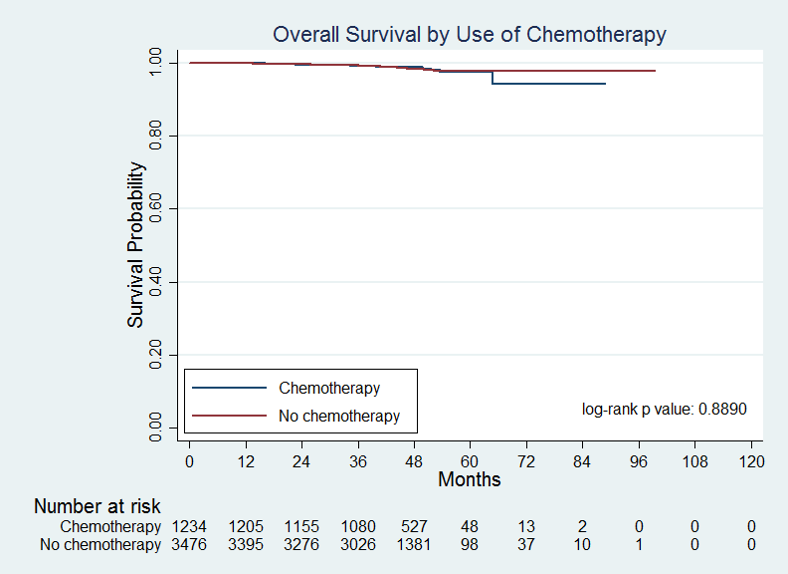

Supplement: Supplementary file 2 — Figure S1. No difference in overall survival by use of chemotherapy for patients diagnosed between 2006 and 2010 with median follow-up of 46.4 months. (TIFF 150 kb) [file 13058_2018_957_MOESM2_ESM.tif]
