# Supplementary material for: Practice patterns and outcomes for patients with node-negative hormone receptor-positive breast cancer and intermediate 21-gene Recurrence Scores
Source: Breast Cancer Res. 2018 Apr 16;20:26. doi: 10.1186/s13058-018-0957-3 (PMC5903005; doi:10.1186/s13058-018-0957-3)
Supplement: Supplementary file 3 — Table S2. Utilization of chemotherapy by year. Chemotherapy use has been decreasing steadily over the years (trend from 2010 to 2013, p < 0.001). (DOCX 14 kb) [file 13058_2018_957_MOESM3_ESM.docx]

Additional file 3: Table S2

Utilization of chemotherapy by year. Chemotherapy use has been decreasing steadily over the years (p for trend from 2010 to 2013 <0.001).

|  | No Chemotherapy | | Chemotherapy | |
| --- | --- | --- | --- | --- |
|  | No. | % | No. | % |
| 2006 | 4 | 80.0 | 1 | 20.0 |
| 2007 | 16 | 69.6 | 7 | 30.4 |
| 2008 | 40 | 64.5 | 22 | 35.5 |
| 2009 | 200 | 69.0 | 90 | 31.0 |
| 2010 | 3244 | 74.2 | 1131 | 25.8 |
| 2011 | 4196 | 77.5 | 1220 | 22.5 |
| 2012 | 4713 | 81.5 | 1069 | 18.5 |
| 2013 | 4932 | 81.6 | 1109 | 18.4 |
