# Supplementary material for: Practice patterns and outcomes for patients with node-negative hormone receptor-positive breast cancer and intermediate 21-gene Recurrence Scores
Source: Breast Cancer Res. 2018 Apr 16;20:26. doi: 10.1186/s13058-018-0957-3 (PMC5903005; doi:10.1186/s13058-018-0957-3)
Supplement: Supplementary file 4 — Table S3. Incidence of individual 21-gene Recurrence Scores from 11 to 25, and the comparative utilization of chemotherapy based on the score. (DOCX 14 kb) [file 13058_2018_957_MOESM4_ESM.docx]

Additional file 4: Table S3

Incidence of individual 21-gene Recurrence Scores from 11 to 25, and the comparative utilization of chemotherapy based on the score.

| Oncotype DX score | Number of patients | Proportion of patients who received chemo |
| --- | --- | --- |
| 11 | 1727 | 3.4% |
| 12 | 1702 | 5.1% |
| 13 | 1812 | 5.8% |
| 14 | 1905 | 7.1% |
| 15 | 1918 | 6.8% |
| 16 | 1940 | 8.6% |
| 17 | 1810 | 11.4% |
| 18 | 1719 | 20.9% |
| 19 | 1467 | 30.7% |
| 20 | 1356 | 37.8% |
| 21 | 1149 | 40.9% |
| 22 | 1038 | 48.3% |
| 23 | 909 | 54.6% |
| 24 | 848 | 59.1% |
| 25 | 691 | 67.9% |
